# Moxifloxacin-Mediated Downregulation of Intestinal P-Glycoprotein Alters the Pharmacokinetics of Dabigatran Etexilate: Mechanistic Insights in Rats and PBPK Model-Informed Dose Optimization

**DOI:** 10.3390/pharmaceutics18081031

**Published:** 2026-08-20

**Authors:** Yuchen Qu, Zhuan Yang, Wen Ma, Peng Xiao, Yani Gu, Jie Pan, Xinyun Zhang, Chen Zhao, Yunli Yu

**Affiliations:** 1 School of Pharmacy, Nanjing Medical University, Nanjing 211166, China; quyuchen1993@163.com (Y.Q.);; 2 Department of Pharmacy, The Second Affiliated Hospital of Soochow University, Suzhou 215004, China; 3 Suzhou Key Laboratory of Translational Research and Application for Radionuclide Therapy and Nuclear Emergency Drugs, The Second Affiliated Hospital of Soochow University, Suzhou 215004, China; 4 Jiangsu Key Laboratory of Radiation Injury Prevention and Hazardous Substance Control, Suzhou 215004, China; 5 College of Pharmaceutical Science, Soochow University, Suzhou 215123, China

**Keywords:** dabigatran etexilate, moxifloxacin, P-gp, PBPK, virtual clinical trial

## Abstract

**Background**: In patients with atrial fibrillation receiving long-term anticoagulation therapy with dabigatran etexilate (DABE), moxifloxacin (MFLX) is frequently coadministered to treat concurrent infections; however, the potential drug–drug interaction (DDI) between these agents remains unclear. Herein, we examined the underlying mechanism by which MFLX attenuates DABE pharmacokinetics in rats; subsequently, we elucidated the DDI in humans by establishing a physiologically based pharmacokinetic (PBPK) model based on these animal data. **Methods**: The 3- and 14-day effects of 40 mg/kg MFLX once daily and secondary bile acid (SBA)-containing dietary intervention on the pharmacokinetic profile of DABE and its active form, dabigatran (DAB), were examined in a rat model. Ileum tissues were harvested to measure the expression of P-glycoprotein (P-gp), pregnane X receptor (PXR), and peroxisome proliferator-activated receptor alpha (PPARα). In addition, we examined the effects of secondary bile acids (SBAs) on P-gp expression and quantified P-gp-mediated DABE efflux transport activity in Caco-2 cells. A PBPK model was used to predict the risk of DAB exposure under this DDI scenario and under combined high-risk conditions, including renal impairment and advanced age. **Results:** Treatment with MFLX for 3 and 14 days inhibited SBA-producing gut microbiota, thereby suppressing the conversion of primary bile acids to SBAs. Concurrently, a marked reduction in intestinal P-gp expression was observed, along with a significant enhancement of the oral bioavailability of DABE. These effects were reversed by SBA-containing diets. In vitro experiments using Caco-2 cells revealed that physiologically relevant concentrations of SBA significantly upregulated P-gp expression and function, whereas MFLX incubation alone showed no direct modulatory effect on these transporters or regulators. PBPK simulation results showed that in vivo exposure of DAB would increase by 41.7%, 104%, 251%, and 115% when coadministered with MFLX alone, with coexisting mild renal impairment, moderate renal impairment, and aging, respectively. **Conclusions**: MFLX increases DAB exposure by reducing SBA-regulated intestinal P-gp function. PBPK simulations suggest a low risk of DDI from MFLX coadministration alone; however, caution is warranted in patients with aging or renal impairment.

## 1. Introduction

Atrial fibrillation (AF) is the most common type of clinical arrhythmia. It is a significant risk factor that increases the likelihood of thromboembolic events in patients [1]. A total of 59.7 million cases of AF were documented worldwide by 2019, and the number has been growing annually [2,3]. Dabigatran etexilate (DABE) is the most widely used novel oral anticoagulant in clinical practice. It exerts its anticoagulant effect by directly inhibiting thrombin activity [4]. Its efficacy in long-term anticoagulation therapy for AF patients has been validated [5]. Because of long-term irregular atrial activity, AF patients may experience reduced cardiac output and pulmonary congestion, which is often accompanied by pulmonary infections during long-term anticoagulation therapy. The results from a domestic clinical study indicate that the incidence of hospital-acquired pneumonia in AF patients is nearly seven-fold higher compared with that in non-AF patients (25.87% vs. 3.63%) [6]. Fluoroquinolones are first-line therapeutic agents for the treatment of pulmonary infections [7]. They are frequently coadministered with DABE. For AF patients undergoing long-term anticoagulation therapy with DABE, the risk of drug overexposure resulting from drug–drug interactions (DDIs) is a significant factor leading to an increased incidence of bleeding events in this patient population [8]. Clinical studies indicate that a 100% increase in DABE systemic exposure can raise the risk of bleeding by 50% to 300% [9,10]. Phase IV clinical trial data for Pradaxa (dabigatran etexilate mesylate) published on the eHealthME website indicate that cotreatment with fluoroquinolones significantly increases the risk of bleeding events associated with DABE overexposure during treatment in AF patients [11]; however, current Pradaxa prescribing information does not specify how to adjust the DABE dosage when coadministered with fluoroquinolones [12], which prevents the adequate assurance of medication safety for these patients.

DABE is an oral prodrug that is rapidly converted to its active form, dabigatran (DAB), following absorption; however, its absorption is incomplete because of poor solubility, which results in an absolute bioavailability of only approximately 3–7% [13]. As an intestinal-specific P-glycoprotein (P-gp) substrate, DABE is subject to intestinal P-gp-mediated efflux, which is a key determinant of systemic DAB exposure [14]. Intestinal P-gp is an important molecule in the intestinal barrier that is regulated by the intestinal microenvironment [15]. The administration of antimicrobial agents can disrupt the intestinal microbiota, thereby disrupting intestinal barrier integrity and affecting the bioavailability of DABE. Chen et al. demonstrated that treatment with the β-lactam antibiotic amoxicillin increased the systemic exposure of oral anticoagulants, likely through alterations in gut microbiota composition [16]; however, related mechanisms remain unclear. Previously, we found that fluoroquinolone use disrupts the intestinal abundance of Firmicutes and Bacteroidetes in rats, resulting in an imbalance in secondary bile acid metabolism [17]. Specifically, secondary bile acids (SBAs), primarily deoxycholic acid (DCA), lithocholic acid (LCA), and their taurine or glycine conjugates, are microbial metabolites that maintain normal intestinal barrier function [18,19]. These bile acids may act on nuclear receptors to regulate the expression of efflux transporters [20,21]. Consequently, gut microbiota imbalance-induced disturbances in bile acid metabolism may contribute to altered DABE exposure following fluoroquinolone administration.

In this study, we determined the effect of moxifloxacin (MFLX), a representative fluoroquinolone antibiotic, on DABE exposure and examined the underlying mechanisms. We first conducted a rat study to investigate the effect of MFLX administration and SBA supplementation on the systemic exposure of DABE and intestinal P-gp expression. Subsequently, we validated the mechanism of action of SBAs using Caco-2 cells. Moreover, Transwell-based transport assays enabled quantitative assessment of the inhibitory effect of MFLX on SBA-mediated P-gp function. A physiologically based pharmacokinetic (PBPK) model was used to predict the pharmacokinetic profile of DABE in AF patients cotreated with MFLX to establish a basis for dose adjustment in these patients.

## 2. Materials and Methods

### 2.1. Chemicals and Reagents

DMEM, fetal bovine serum, streptomycin, and penicillin G were purchased from Procell (Wuhan, China). MFLX (98%) was obtained from Shanghai Macklin Biochemical Co., Ltd. (Shanghai, China). DCA (98%), LCA (95%), DABE (98%), DAB (≥98%), and d4-rivaroxaban were obtained from Aladdin (Shanghai, China). The PXR inhibitor (resveratrol) and PPARα inhibitor (GW6471) were purchased from MedChemExpress (Monmouth Junction, NJ, USA). Acetonitrile (high-performance liquid chromatography grade) was obtained from Merck KGaA (Darmstadt, Germany). Ammonium acetate was purchased from Macklin (Shanghai, China).

### 2.2. Animals and Treatment

A rat dosing study was conducted to investigate the effect of MFLX coadministration on the pharmacokinetic profile of DABE and to elucidate the underlying mechanisms. Male 8-week-old Wistar rats were obtained from B&K Laboratory Animal Technology (Shanghai, China). After a one-week acclimation period under controlled environmental conditions (12 h light/dark cycle, 21 °C ± 2 °C) with free access to food and water, the animals were randomly assigned to one of three groups: the CT group, the MFLX group, or the MFLX + SBA group. Each group consisted of five rats, balanced by body weight. The MFLX and MFLX + SBA groups were treated with MFLX (40 mg/kg) once daily via gavage for 3 and 14 days, respectively. The rats in the CT group received the vehicle (0.25% CMC-Na solution) instead. The CT and MFLX groups were provided a standard chow diet, whereas the MFLX + SBA group was provided the standard diet supplemented with 10 mmol/kg each of deoxycholic acid and lithocholic acid. This SBA supplementation was designed to counteract the suppression of endogenous SBA production in the gut induced by MFLX treatment, thereby restoring the intestinal SBA pool to evaluate whether SBA deficiency mediates the pharmacological effects of MFLX. DABE (8 mg/kg) was administered to the rats by oral gavage on day 3 and day 14 to evaluate the short-term and long-term effects of MFLX, respectively, following an overnight fast. All animal experiments were approved by the Animal Ethics Committee of Soochow University (SUDA20241121A03).

### 2.3. Serum and Tissue Sample Collection

Orbital blood samples were collected from rats at baseline (0 min), 20 min, 40 min, 1 h, 1.5 h, 2.5 h, 4 h, 6 h, 8.5 h, and 12 h following the oral administration of DABE. Serum samples were collected by centrifugation at 2500× *g* for 10 min at 4 °C and stored at −80 °C for the pharmacokinetic study. After collecting the pharmacokinetic samples, the rats were anesthetized with an intraperitoneal injection of sodium pentobarbital (60 mg/kg). The ileum tissues were excised and stored at −80 °C for subsequent testing.

### 2.4. LC–MS/MS Analysis

Quantitative determination of DAB was done using liquid chromatography and tandem mass spectrometry (LC–MS/MS) with d4-rivaroxaban as the internal standard (IS). The separation of DAB was performed on a Waters XTerra RP18 column (4.6  ×  150 mm, 5 µm) using an API4000 system (AB Sciex, Framingham, MA, USA) operated in positive ion mode. The mobile phase contained 5 mM aqueous ammonium acetate (A) and acetonitrile (B). The flow rate was 0.6 mL/min, and the injection volume was 10 µL. The gradient program was as follows: 30% A from 0 to 3.8 min, 5% A at 4 min, 5% A from 4 to 5.5 min, and 30% A from 5.6 to 8 min. The retention times for DAB and IS were 2.19 min and 3.01 min, respectively. Acquisition was performed in multiple reaction monitoring mode. The MS/MS parameters for DAB were as follows: MS/MS transition, *m*/*z* 472.5 (the precursor ion)/289.1 (the product ion); declustering potential, 84 V; and collision energy, 36 V. The MS/MS parameters for IS were as follows: MS/MS transition, *m*/*z* 440.4 (the precursor ion)/145.3 (the product ion); declustering potential, 95 V; and collision energy, 37 V. All MS spectra were acquired and analyzed using the AB Sciex API 4000 system (AB Sciex, Framingham, MA, USA).

Serum samples were prepared for LC–MS/MS analysis as follows: 100 µL of 10 µg/mL IS in 100% acetonitrile was added to 50 µL of serum in a 1.5-mL Eppendorf tube. The mixture was vortexed for 1 min and centrifuged at 18,000× *g* for 10 min at 4 °C. The resulting supernatant was collected for LC–MS/MS injection. For cell culture media samples, 50 µL of medium was similarly treated with 100 µL of the same IS solution before analysis. Detailed LC-MS/MS analysis methods are provided in the Supplementary Material.

### 2.5. RNA Extraction and Real-Time PCR

Total RNA was extracted from frozen tissues or cell lines using TRIzol reagent and a standard phenol-chloroform method. Next, cDNA was synthesized from 2 μg of total RNA using a reverse transcription kit (ABM, Cat#G490). Real-time quantitative PCR was performed on an Applied Biosystems QuantStudio Dx platform (Thermo Fisher Scientific, Waltham, MA, USA) with universal SYBR Green (APE × BIO, Houston, TX, USA) as the fluorescent dye. The primer sequences are listed in Supplementary Table S1.

### 2.6. Western Blot Analysis

Total protein was extracted from ileum samples and cell lines using RIPA buffer. Protein concentration was determined using the BCA assay (Biosharp, Beijing, China). The extracts were subjected to sodium dodecyl sulfate-polyacrylamide gel electrophoresis (SDS-PAGE), and the proteins were transferred onto a polyvinylidene difluoride (PVDF) membrane using a wet transfer system. After blocking for 2 h at room temperature, the membranes were incubated overnight at 4 °C with the following primary antibodies: P-gp (Abcam, Cambridge, UK, ab170904), PXR (Proteintech, Rosemont, IL, USA, 67912-1-Ig), PPARα (Proteintech, Rosemont, IL, USA, 66826-1-Ig), Histone H3 (ABclonal, Woburn, MA, USA, A2348), and β-actin (Proteintech, Rosemont, IL, USA, 66009-1-Ig). The membranes were washed three times with TBST (Sangon Biotech, Shanghai, China) for 10 min each, followed by incubation with secondary antibodies (Proteintech, Rosemont, IL, USA) for 1 h at room temperature. After washing with TBST, the protein bands were visualized using an enhanced chemiluminescence (ECL) reagent and quantified with ImageJ software (version 1.53).

### 2.7. Cell Lines

Caco-2 cells (RRID: CVCL_0025) were obtained from the National Collection of Authenticated Cell Cultures (Shanghai, China) and cultured in DMEM supplemented with 20% FBS and 1% penicillin/streptomycin at 37 °C in a humidified incubator containing 5% CO_2_.

### 2.8. Transport Studies

Transport studies were performed in Caco-2 cell monolayers to quantitatively characterize the effect of SBAs on P-gp-mediated DABE transport and to obtain key parameters for PBPK modeling. Caco-2 cells were seeded on polyester membranes in Transwell filters (Corning, NY, USA) at a density of 2 × 10^5^ cells/mL and incubated for 21 days to establish a monolayer. Monolayer integrity was monitored every 2–3 days by measuring transepithelial electrical resistance (TEER) using a Millicell-ERS instrument (Millipore, Billerica, MA, USA). Before the transport studies, the cells were preincubated with physiologically relevant concentrations of SBA (500 μM DCA + 50 μM LCA) for 6, 24, 48, and 72 h. To monitor the transport of DABE from the apical (AP) to the basolateral (BL) compartment, 0.5 mL of a 2 μM DABE solution was added to the AP side and 1.5 mL of HBSS was added to the BL side. Following sample addition, the cell culture plate was incubated at 37 °C, 5% CO_2_, and saturated humidity for 120 min. The apparent permeability coefficient (P_app_) was calculated using the following equation: P_app_ = (dQ/dt)/(A × C0), where dQ/dt is the rate of drug transport per unit time, A is the surface area of the Transwell membrane, and C0 is the initial concentration of DABE in the donor compartment.

### 2.9. Pharmacokinetic Analysis and Statistical Analysis

PBPK modeling and simulation were performed using GastroPlusTM (version 9.9; Simulation Plus Inc., Lancaster, CA, USA). The detailed procedures for basic PBPK module construction have been described in our previous work [22]. Briefly, the model was built upon an extensive literature survey to compile representative compound-specific data and clinical pharmacology studies, including physicochemical properties, in vitro findings, and absorption, distribution, metabolism, and excretion parameters. The predictive performance of the model was evaluated using published clinical trial data obtained from healthy subjects after oral administration of DABE at doses of 150 mg and 300 mg. In addition, the predictive capability of the P-gp-mediated DDI was verified using clinical data from clarithromycin coadministration studies. The detailed drug-specific parameters of DABE and DAB are listed in Supplementary Table S2. In this study, we first incorporated the functional variation of P-gp derived from in vitro transport studies to construct a virtual patient population under the DDI scenario. We then focused on two high-risk patient subgroups, the elderly and those with mild-to-moderate renal impairment, to validate the model’s predictive performance for DAB in these populations. Finally, simulation was performed in patients receiving MFLX coadministration with concurrent elderly and renal impairment to characterize the safety risk attributable to elevated systemic DAB exposure in these high-risk populations.

Statistical analysis was performed using GraphPad Prism software, version 10.1.2 (GraphPad Software, La Jolla, CA, USA). Comparisons were performed using an unpaired two-tailed Student’s *t*-test. Noncompartmental pharmacokinetic analysis was performed using MATLAB R2023b.

## 3. Results

### 3.1. MFLX Significantly Increases the Systemic Exposure of DABE in Wistar Rats

**Figure 1:**
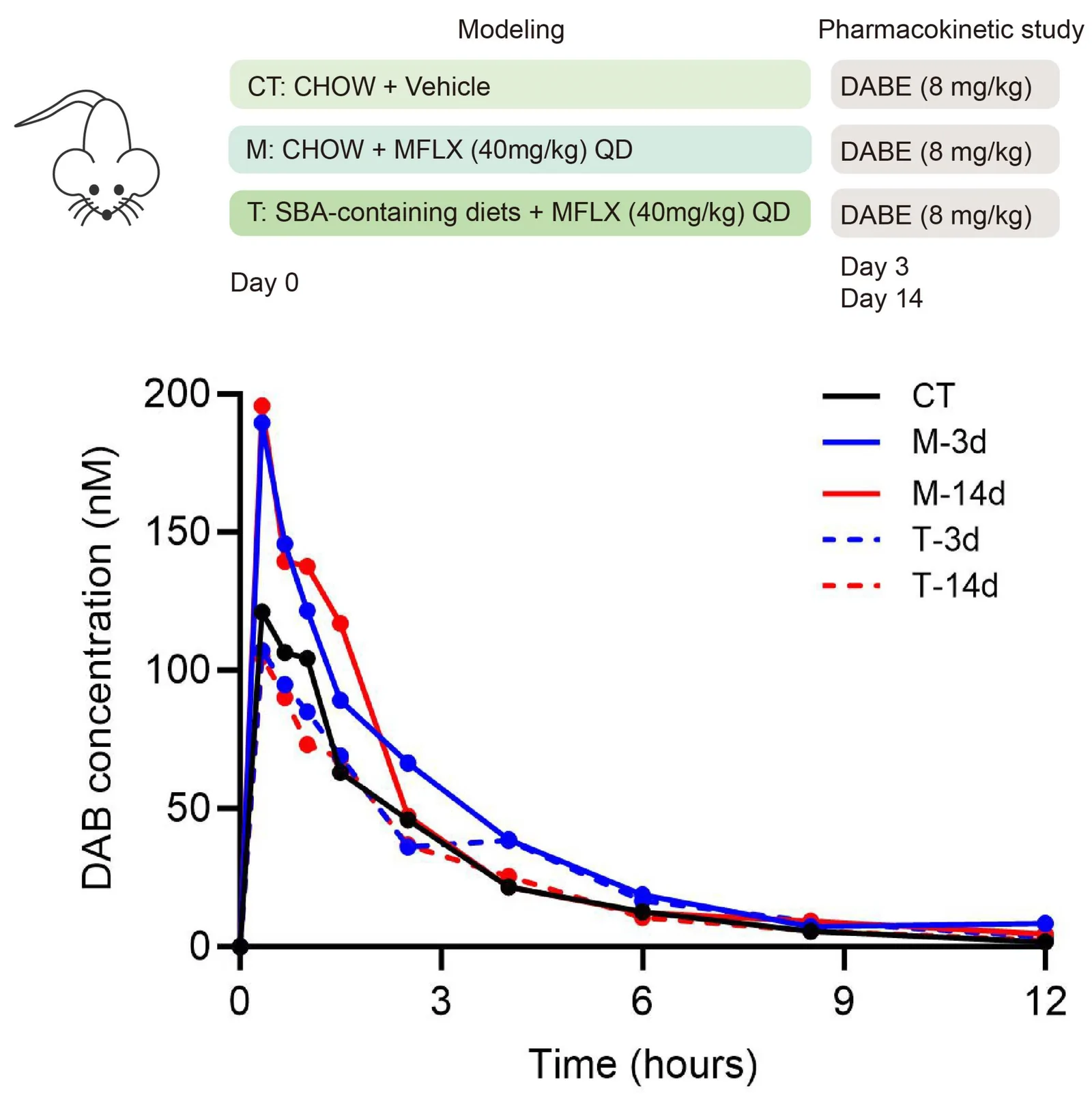
Serum concentration–time profiles for DAB in rats. Comparison among the control group (black solid line), MFLX-treated groups for 3 and 14 days (blue and red solid lines), and MFLX-treated groups for 3 and 14 days with SBA-containing diets (blue and red dashed lines). CHOW: standard chow diets; CT: control group; M-3d: MFLX intervention for 3 days; M-14d: MFLX intervention for 14 days; T-3d: MFLX intervention combined with SBA supplementation for 3 days; M-14d: MFLX intervention combined with SBA supplementation for 14 days.

MFLX pretreatment altered the pharmacokinetic profile of DAB following DABE administration in Wistar rats (Figure 1). The noncompartmental pharmacokinetic analysis revealed that the Cmax of DAB increased by 41.4% and 53.0%, whereas the AUC_0–12h_ increased by 47.8% and 34.5% following 3- and 14-day MFLX pretreatment, respectively. SBA-containing diets reversed abnormally elevated DAB exposure caused by MFLX administration (Table 1).

### 3.2. Increased DAB Exposure Is Associated with Impaired P-gp Function in the Ileum

**Figure 2:**
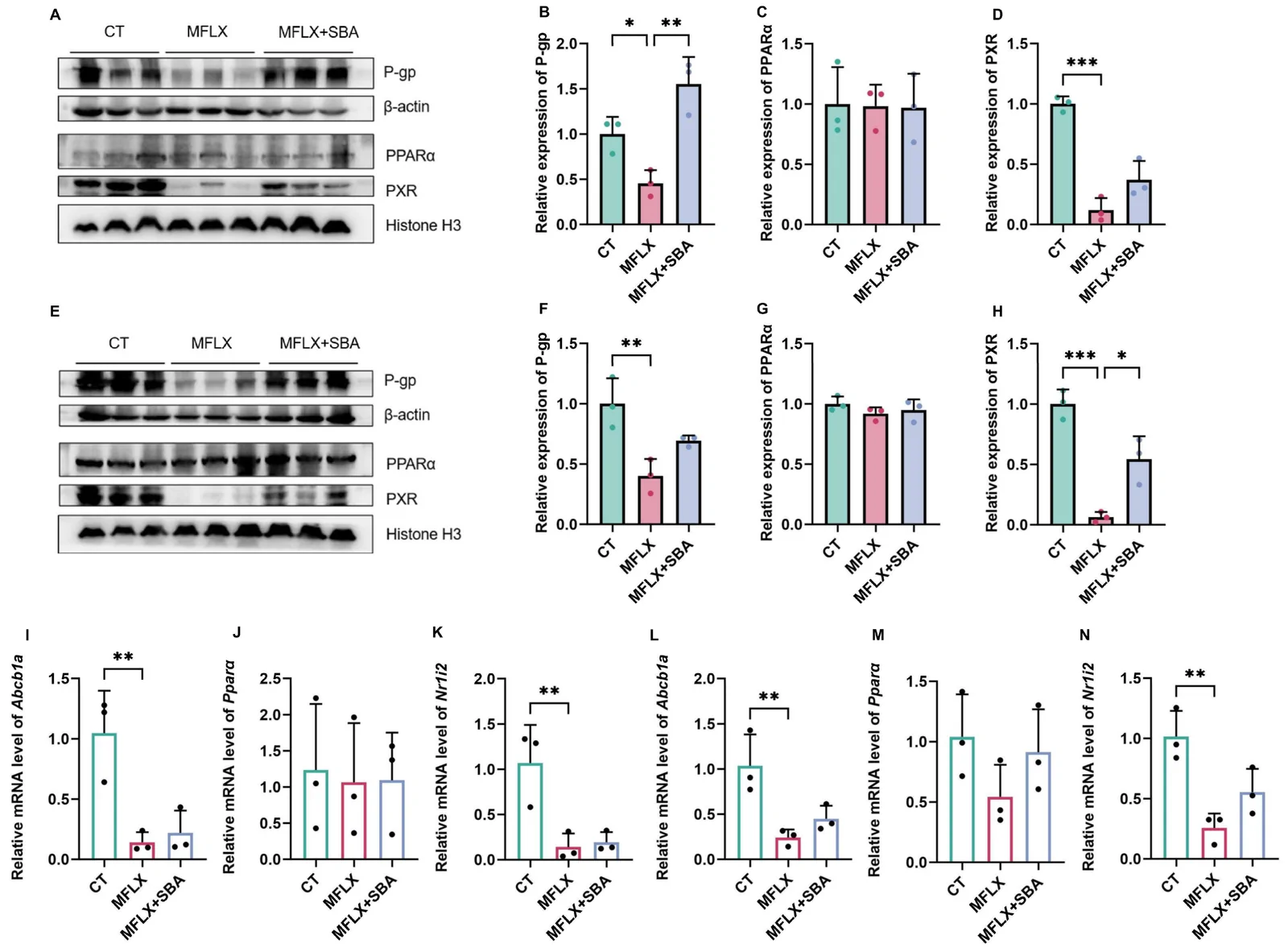
Expression of P-gp, PXR, and PPARα in rat ileum tissue after 3 and 14 days of DABE administration. (**A**–**H**) Relative expression of P-gp, PPARα, and PXR proteins in the rat ileum after 3 days (**A**–**D**) and 14 days (**E**–**H**) of DABE administration. (**I**–**N**) Relative expression of *Abcb1a*, *Pparα*, and *Nr1i2* mRNA in the rat ileum after 3 days (**I**–**K**) and 14 days (**L**–**N**) of DABE administration. The data are presented as the mean ± SD; * *p* < 0.05, ** *p* < 0.01, *** *p* < 0.001.

DABE is a specific substrate of intestinal P-gp. Alterations in intestinal P-gp function can affect the systemic exposure of DABE. Figure 2 shows the protein and mRNA expression levels of intestinal P-gp in rats following MFLX administration and SBA-containing dietary intervention. MFLX significantly downregulated intestinal P-gp expression (encoded by Abcb1a) after 3 and 14 days of DABE administration. After an SBA-containing dietary intervention, P-gp protein and mRNA upregulation were reversed. In the intestine, SBA regulates nuclear receptors, which in turn regulate the expression of P-gp. The protein and mRNA levels of PXR exhibited the same trend as P-gp; however, PPARα protein expression did not significantly change.

### 3.3. SBA Affects P-gp Expression and DABE Transport in Caco-2 Cells

**Figure 3:**
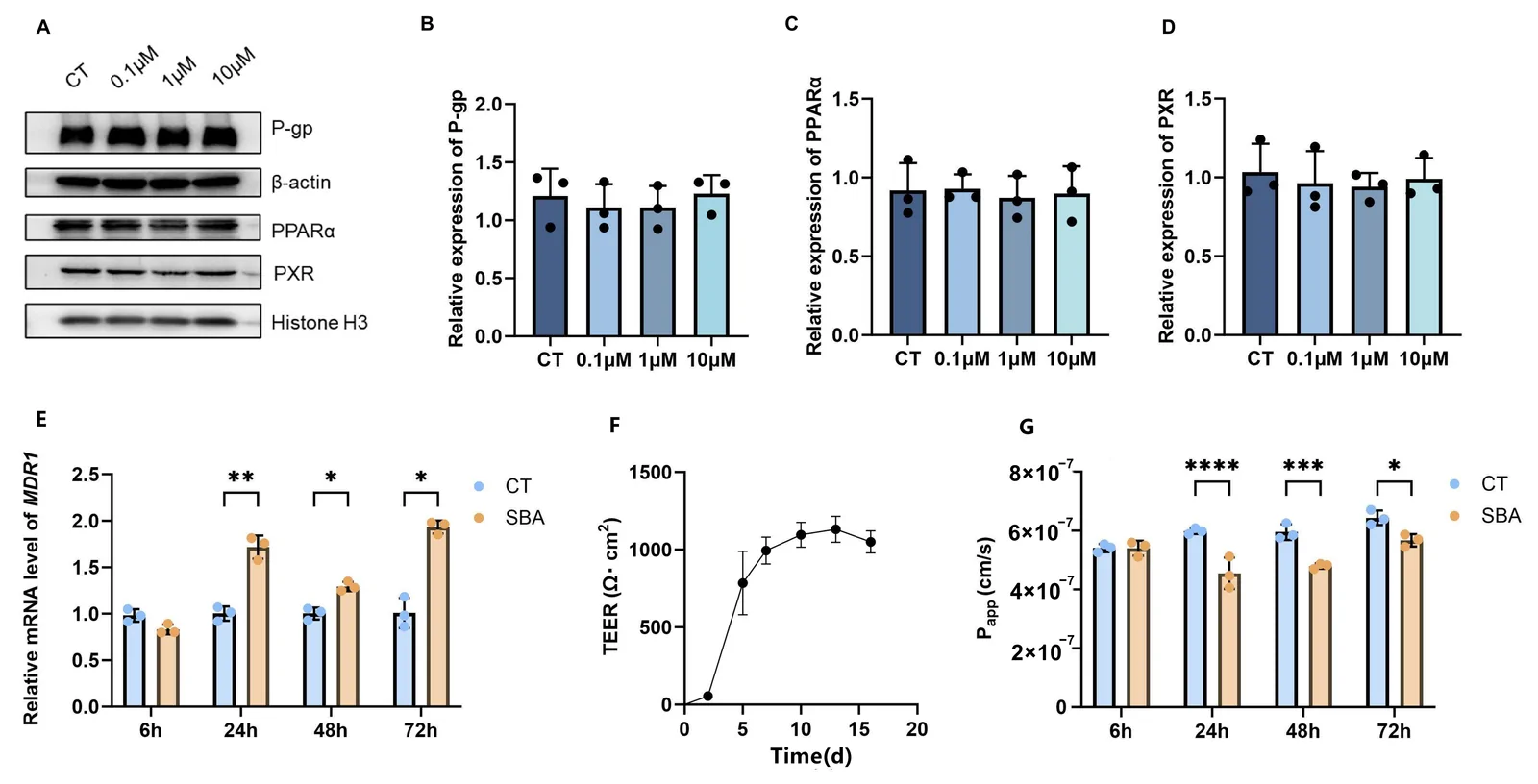
MFLX and SBA effect on target gene expression and transport function of Caco-2 cells. (**A**–**D**) Relative protein expression of P-gp, PPARα, and PXR in Caco-2 cells following treatment with 0.1, 1, and 10 μM MFLX for 24 h. (**E**) Relative mRNA expression of *MDR1* following treatment with or without physiologically relevant concentrations of SBA for 6, 24, 48, and 72 h. (**F**) Transepithelial electrical resistance (TEER) values of the Caco-2 monolayers. (**G**) P_app_ of DABE using Caco-2 monolayers following treatment with or without physiologically relevant concentrations of SBA for 6, 24, 48, and 72 h. Data are presented as the mean ± SD, * *p* < 0.05, ** *p* < 0.01, *** *p* < 0.001, **** *p* < 0.0001.

Direct incubation of MFLX with Caco-2 cells at 0.1, 1, and 10 μM did not affect the expression of P-gp, PXR, or PPARα (Figure 3A–D). Following treatment with physiologically relevant concentrations of SBA, no significant change in P-gp expression was observed at 6 h; however, marked increases were evident at 24, 48, and 72 h (Figure 3E). In contrast to the observation in the rat ileum, SBA incubation in Caco-2 cells not only altered PXR expression but also significantly increased PPARα expression (Supplementary Figure S1).

Caco-2 monolayers with confirmed barrier integrity were successfully established to validate P-gp-mediated DABE efflux. When Caco-2 cells were cultured for 21 days, they were tightly connected without gaps. Their development was characterized by a significant increase in TEER beginning on day 7 post-seeding. The TEER continued to rise until day 18, after which it stabilized above 1000 Ω·cm^2^. Subsequent experiments used these mature monolayers maintained at a TEER > 1000 Ω·cm^2^ (Figure 3F). The effects of SBA on DABE transport were assessed. SBA pretreatment exhibited a time-dependent effect on the P_app_ value. No change was observed at 6 h, whereas significant decreases of 23.9%, 19.4%, and 11.9% were evident at 24, 48, and 72 h, respectively (Figure 3G).

**Figure 4:**
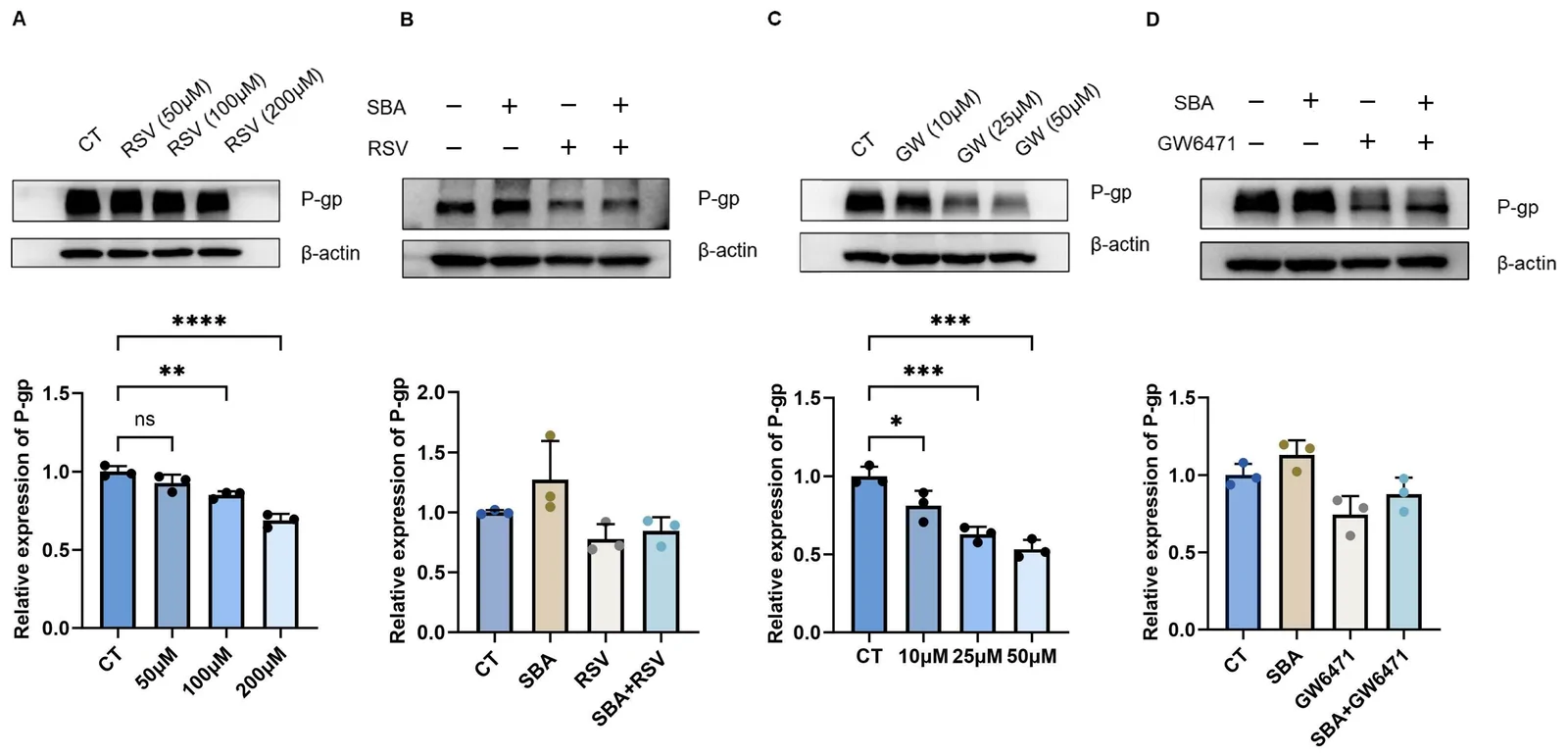
Effects of PXR and PPARα inhibitors on SBA-induced P-gp upregulation in Caco-2 cells. Relative P-gp protein expression following treatment with various concentrations of the PXR inhibitor resveratrol (**A**) and the PPARα inhibitor GW6471 (**B**). Effect of physiologically relevant concentrations of SBA combined with 200 μM of resveratrol (**C**) or 25 μM of GW6471 (**D**) on P-gp expression. RSV, resveratrol; GW, GW6471. Data are presented as the mean ± SD, * *p* < 0.05, ** *p* < 0.01, *** *p* < 0.001, **** *p* < 0.0001.

The effects of physiologically relevant concentrations of SBA alone or in combination with the PXR inhibitor resveratrol or the PPARα inhibitor GW6471 on P-gp expression were examined in Caco-2 cells. Resveratrol and GW6471 suppressed dose-dependent P-gp expression (Figure 4A,C). When co-incubated with SBA, inhibition of either PXR or PPARα was able to block the upregulation of P-gp (Figure 4B,D). Notably, PXR inhibition had a more pronounced effect, completely eliminating the SBA-induced P-gp upregulation.

### 3.4. Development and Verification of the Prediction Model for DABE Exposure in the Virtual DDI Patient

Virtual patients receiving MFLX coadministration were first constructed using the P-gp function parameter derived from an in vitro–in vivo extrapolation method. Specifically, the P-gp function was decreased by 13% to reflect P_app_ variation observed in transport studies following a 72 h incubation with physiologically relevant concentrations of SBA. The modeling simulation results suggested that the MFLX combination increased the steady-state exposure of DAB by 41.7% compared with the general patient.

**Figure 5:**
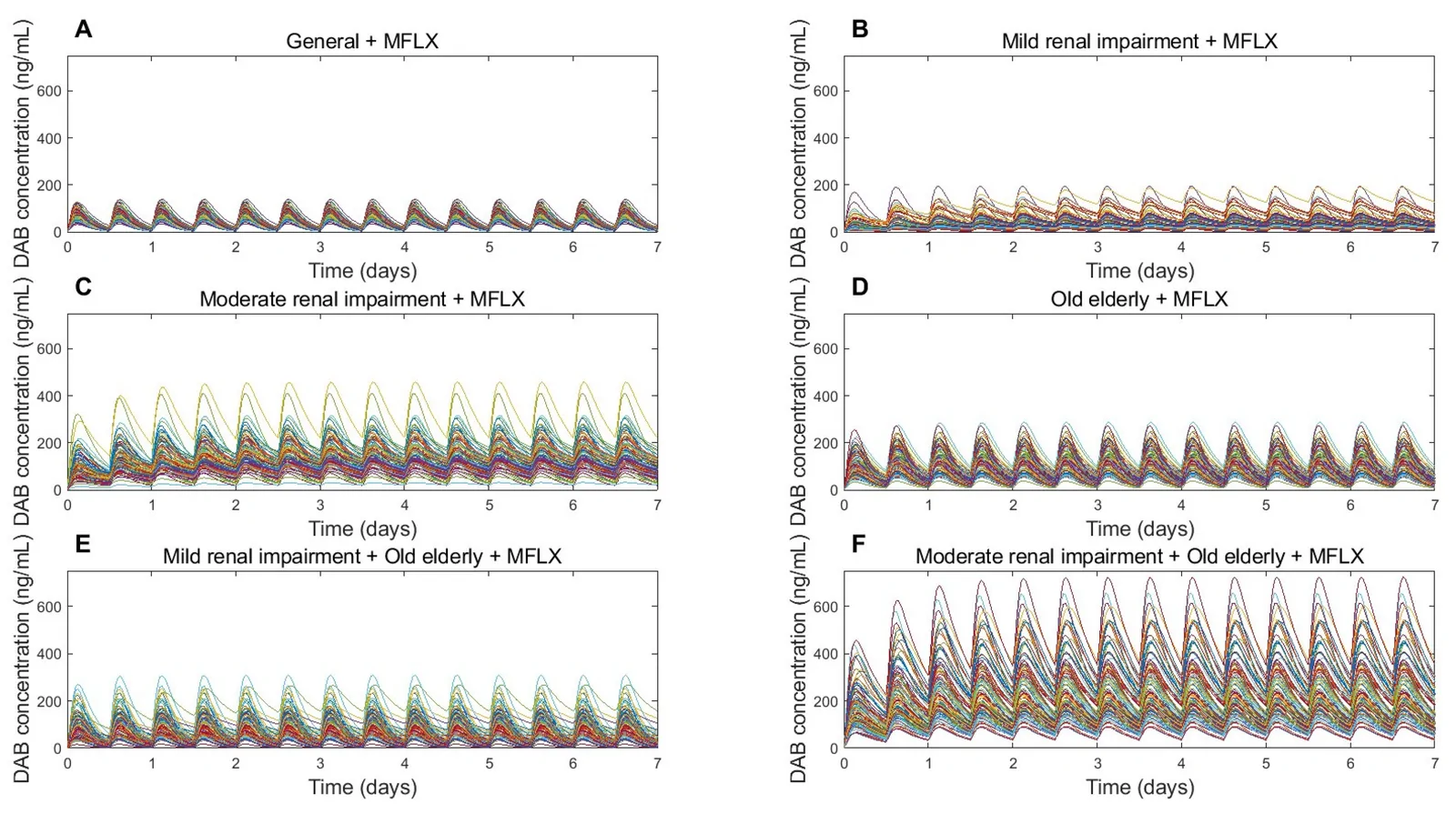
Plasma DAB concentration for a sample simulation run with 500 virtual patients treated with DABE 110 mg twice-daily in combination with MFLX. (**A**) General patients, (**B**) patients with mild renal impairment, (**C**) patients with moderate renal impairment, (**D**) old elderly patients, (**E**) old elderly patients with mild renal impairment, (**F**) old elderly patients with moderate renal impairment.

**Figure 6:**
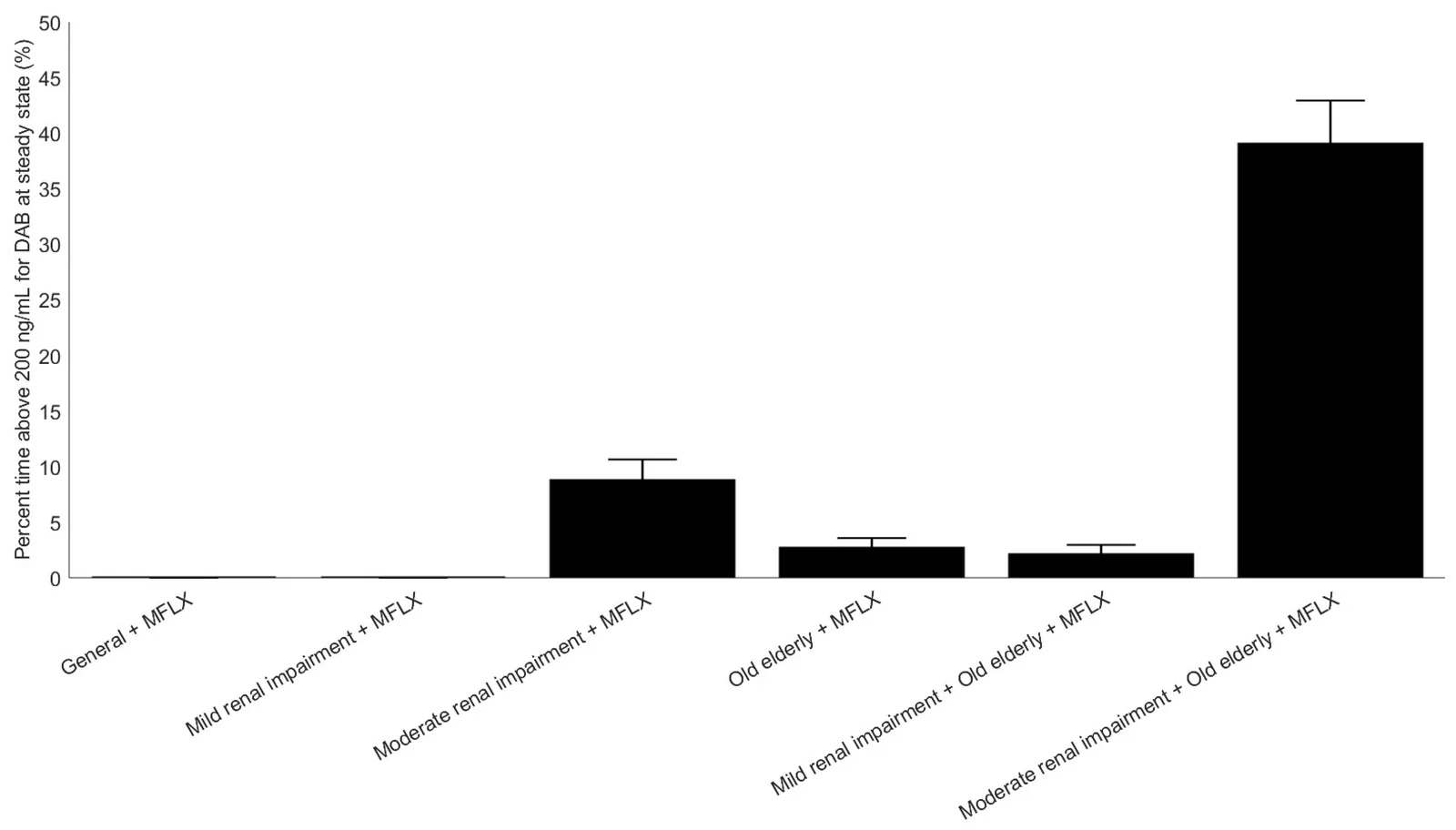
Percent time above 200 ng/mL for plasma DAB concentration at steady state. Data are presented as the mean ± SE.

In humans, 80% of DAB is excreted via glomerular filtration, and renal function is a key factor affecting its systemic exposure [23]. As shown in Supplementary Figure S2, our model adequately fit the pharmacokinetic profile of DAB in patients with mild-to-moderate renal impairment. Moreover, aging represents an independent risk factor for elevated systemic exposure to DAB, independent of renal impairment. The risk of major bleeding with DABE 150 mg twice daily in patients aged ≥ 75 years was approximately 1.4-fold higher than that in patients aged < 75 years [24]. Virtual elderly patients were constructed via an investigator-initiated real-world trial to calibrate the effect of P-gp variability in this population [23]. Simulated steady-state exposure parameters of DAB following administration of 110 mg twice-daily DABE in specific patient populations are listed in Table 2. Compared with general patients, coadministration with MFLX resulted in increased DAB exposure of 104%, 251%, and 115% in patients with mild renal impairment, moderate renal impairment, and elderly patients, respectively. In this DDI scenario, exposure increases of 186% and 134% were observed in old-elderly patients with concomitant mild or moderate renal impairment, respectively. Furthermore, a virtual population was generated to examine DAB exposure at the population level. To achieve DAB PK variability similar to that observed in our previous real-world study [23], the virtual population incorporated 30% variability in P-gp function and physiological sensitivity parameters. The recommended 110 mg twice-daily DABE regimen combined with MFLX alone does not result in a high risk of DAB exposure (Figure 5). The risk increases in patients with decreasing renal function, particularly among elderly patients. As indicated in the product information for DABE (Pradaxa^®^), steady-state DAB levels above 200 ng/mL are associated with increased bleeding risk [12]. Using this value as an acceptable threshold, our simulation demonstrated that a 110 mg twice-daily regimen is safe for both general patients and those with mild renal impairment under MFLX coadministration. Percent time above averaged 8.8% (2.1 h per day) in patients with moderate renal impairment, and this proportion markedly increased to 39.1% (9.4 h per day) in the old-elderly population with moderate renal impairment (Figure 6).

## 4. Discussion

Direct oral anticoagulants represent the most recent class of anticoagulant agents for the treatment of thromboembolic diseases [25]. Of these, DABE was the first approved direct oral anticoagulant and is now widely recommended as first-line therapy for reducing the risk of stroke and systemic embolism in patients with nonvalvular atrial fibrillation [26]. Although DABE has a wider therapeutic window compared with warfarin, exposure-related bleeding risk remains a serious complication and a significant cause of poor prognosis [9,10]. Thus, the rational application of DABE is important for ensuring long-term anticoagulation efficacy and safety in these patients.

The correlation between systemic exposure of DAB and intestinal P-gp function was established in our previous studies [22,24]. In the present study, we focused on a potential perpetrator drug, MFLX, to elucidate DDI between DABE and fluoroquinolones. First, we demonstrated that the bioavailability of DABE was increased after 3- and 14-day MFLX treatment in rats, which was accompanied by a significant reduction in intestinal P-gp expression. Consistently, we observed a synchronous decrease in the expression of the nuclear receptor PXR, which is a transcriptional regulator of P-gp [27]. In vitro incubation experiments using Caco-2 cells demonstrated that MFLX does not directly affect P-gp or PXR expression. This suggests that MFLX may impair gut microbiota homeostasis and alter the production of intestinal microbial metabolites, thereby indirectly affecting P-gp function.

Bile acids are endogenously synthesized from cholesterol and are primarily present in bile. They play an important role in maintaining intestinal homeostasis [18,28]. Bile acids may be divided into PBAs and SBAs. PBAs are synthesized in the liver and secreted into the small intestine, where they are metabolized into SBAs, such as DCA and LCA, by SBA-producing gut microbiota [29]. Our recent studies indicated that short-term antibiotic treatment inhibits the conversion of PBAs to SBAs in the rat intestine. MFLX intervention suppressed Ruminococcaceae, which resulted in decreased 7α-dehydroxylase activity and SBA formation [30]. Specifically, fecal DCA concentrations decreased more than 10-fold from a baseline of approximately 50 nmol/g following 3 days of MFLX administration. A comparable reduction was also observed for LCA, which had a baseline concentration approximately one-tenth that of DCA. SBAs serve as endogenous ligands for multiple nuclear receptors, including FXR, PPAR, and PXR, among others [31]. In the present study, DAB exposure was significantly increased following MFLX administration, whereas dietary intervention with SBA-containing treatment reversed this effect. Consistently, P-gp expression was markedly downregulated in the rat ileum, along with reduced PXR expression. To determine whether SBA is involved in this process, Caco-2 cells were incubated with various concentrations of MFLX and physiologically relevant concentrations of SBA. MFLX alone did not affect P-gp expression in Caco-2 cells; however, physiologically relevant concentrations of SBA significantly increased its expression. Notably, coincubation with a PXR inhibitor abolished the SBA effect. These results further support our hypothesis that MFLX reduces P-gp expression by inhibiting SBA-producing gut microbiota, thereby decreasing SBA production and intestinal PXR activation. It was also observed that SBA incubation upregulated PPARα expression in Caco-2 cells, whereas this effect was not observed in the rat ileum. This discrepancy between the in vitro and in vivo models may be attributed to the presence of gut microbiota or inherent species differences. In addition, we established Caco-2 cell monolayers to quantify P-gp-mediated DABE efflux transport activity under normal physiological SBA concentrations. The results suggested that incubation with SBA for 24, 48, and 72 h significantly reduced the P_app_ value of Caco-2 layers, with the 24 h treatment being the most effective.

To determine the effect of the MFLX combination on patients at high risk for DABE exposure, we established a PBPK model and conducted a series of simulations. First, we constructed a virtual patient population to simulate the effects of short-term MFLX treatment using experimental results from Caco-2 cell monolayers with or without 72 h of physiologically relevant SBA preincubation. Using our previously validated PBPK model of DABE and DAB, the simulation results indicated that the AUC of DAB increased by 41.7% in this subpopulation. In real-world clinical settings, in addition to the use of concomitant medications, these patients often present with additional high-risk factors, such as aging and renal impairment. Therefore, we predicted the plasma concentration–time profile of DAB based on the combined effect of concomitant MFLX therapy and high-risk physiological factors. The results indicated that, compared with the general population, the AUC of DAB increased by 115%, 104%, and 251% when MFLX was coadministered in the presence of aging, mild, and moderate renal impairment, respectively. The simulation results from virtual patients further confirmed this conclusion. When renal impairment and aging occur, the risk of DABE from high exposure to concomitant medications further increases. Thus, the bleeding risk associated with high DAB exposure warrants attention in these populations. We are currently preparing to conduct an investigator-initiated clinical trial to validate this DDI scenario in clinical practice.

## 5. Conclusions

**Figure 7:**
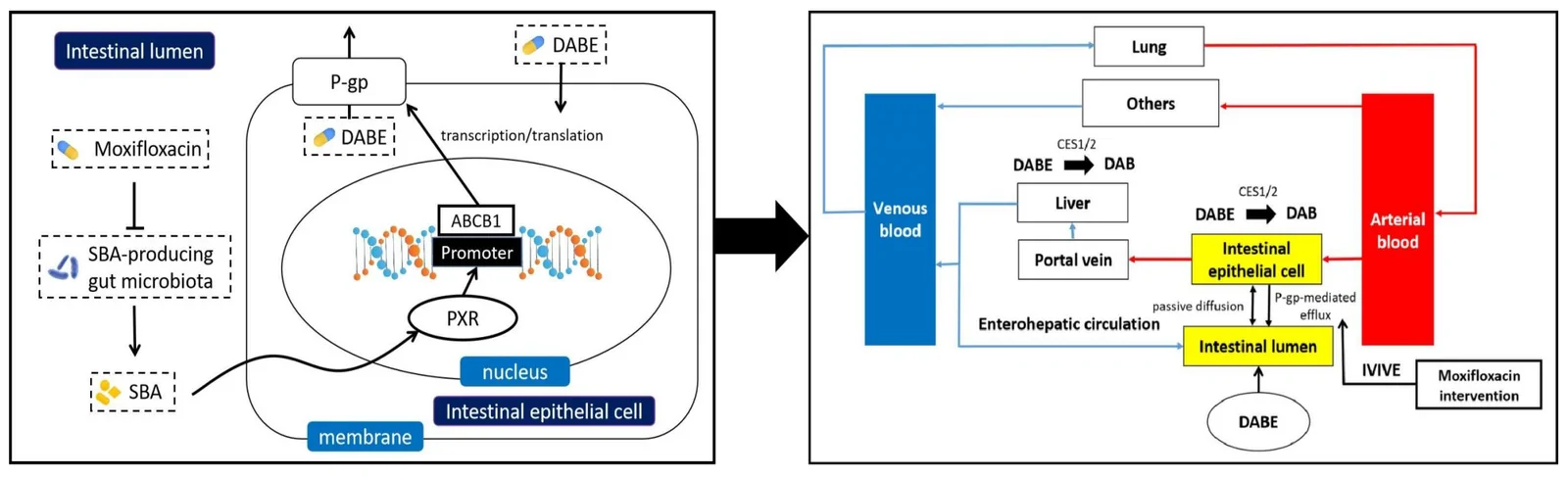
Schematic illustration of the research framework.

In this study, we investigated the mechanisms by which MFLX impairs P-gp function and increases DAB exposure in rats and Caco-2 cells, and developed a PBPK model to predict DAB exposure in different patient groups receiving concomitant MFLX (Figure 7). Our research suggests that: (1) MFLX increases DAB exposure by reducing SBA-regulated intestinal P-gp function, an effect that is mediated by the suppression of SBA-producing gut microbiota. (2) PBPK simulation results indicated that MFLX coadministration alone posed a relatively low safety risk of elevated DAB exposure; however, the potential safety risk associated with this DDI scenario should be carefully considered in elderly patients or those with renal impairment.

## Figures and Tables

**Table 1:** Noncompartmental pharmacokinetic analysis results of DAB following a single oral administration of 8 mg/kg DABE in Wistar rats (n = 5 per group).

Group	Pharmacokinetic Parameters, Mean (SD)						
	t_1/2_ (h)	T_max_^a^ (h)	C_max_ (nM)	AUC_0–12h_ (nM·h)	AUC_inf_ (nM·h)	V_z_ (L/kg)	CL/F (L/h/kg)
C	1.95 (0.402)	0.33 (0.33–0.67)	137 (55.2)	310 (69.9)	316 (75.6)	72.0 (10.2)	26.6 (7.14)
M-3d	2.96 (1.76)	0.33 (0.33–1.00)	194 (124)	458 (186)	510 (174)	74.0 (46.3)	17.4 (6.96)
T-3d	2.52 (0.681)	0.33 (0.33–0.67)	107 (14.2)	338 (86.5)	352 (93.7)	83.4 (20.0)	23.9 (6.15)
M-14d	2.86 (1.19)	0.33 (0.33–1.50)	210 (101)	417 (176)	438 (194)	78.2 (17.6)	22.0 (11.1)
T-14d	2.23 (0.763)	0.33 (0.33–1.50)	106 (28.8)	282 (62.6)	291 (68.4)	89.9 (31.5)	28.5 (5.96)

C: control group; M: MFLX-treated groups; T: MFLX-treated groups with SBA-containing diets. ^a^ Data are presented as median (range). t_1/2_, elimination half-life; T_max_, time to maximum serum concentration; C_max_, maximum serum concentration; AUC_0–12h_, area under the serum concentration–time curve from zero to 12 h; AUC_inf_, area under the serum concentration-time curve from zero to infinity; Vz, volume of distribution; CL/F, apparent clearance.

**Table 2:** Steady-state PK parameters of DAB in different types of virtual typical patients after 110 mg twice-daily DABE administration.

Group	AUC_τ.ss_ (ng·h/mL)	C_max,ss_ (ng/mL)	C_trough,ss_ (ng/mL)
General	450.1	69.16	9.95
General + MFLX	638.0	89.23	18.84
Mild renal impairment + MFLX	916.4	113.59	39.67
Moderate renal impairment + MFLX	1580	172.93	84.95
Old elderly + MFLX	967.6	125.05	32.21
Mild renal impairment + Old elderly + MFLX	1289	152.28	63.71
Moderate renal impairment + Old elderly + MFLX	1992	218.20	107.50

AUC_τ.ss_, area under the plasma concentration–time curve during one dosing interval at steady-state; C_max,ss_, maximum plasma concentration at steady-state; C_trough,ss_, trough plasma concentration at steady-state.

## Data Availability

The original contributions presented in the study are included in the article/Supplementary Material; further inquiries can be directed to the corresponding authors.

## Supplementary Materials

The following supporting information can be downloaded at: https://www.mdpi.com/article/10.3390/pharmaceutics18081031/s1. Ref. [32] was cited in Supplementary Material.
