# Supplementary material for: Moxifloxacin-Mediated Downregulation of Intestinal P-Glycoprotein Alters the Pharmacokinetics of Dabigatran Etexilate: Mechanistic Insights in Rats and PBPK Model-Informed Dose Optimization
Source: Pharmaceutics. 2026 Aug 20;18(8):1031. doi: 10.3390/pharmaceutics18081031 (PMC13516782; doi:10.3390/pharmaceutics18081031)
Supplement: Supplementary file 1 [file pharmaceutics-18-01031-s001.zip › Supplemental Materials S1.pdf]

Supplemental Materials

Supplemental Tables

Table S1. List of primers used in this study for RT-PCR analysis.

Species	Gene	Forward primer (5'-3')	Reverse primer (3'-5')
Rat	Abcb1a	TTCAACTATCCCACCCGACCCA	CTGTACTCTTCCCGCAGCCACT
	PXR	GACGGCAGCATCTGGAACTAC	TGATGACGCCCTTGAACATG
	PPARα	TACCTGTGAACACGATCTGA	GCTAGTCTTTCCTGCGAGTA
	β-actin	CACCCGCGAGTACAACCTTC	CCCATACCCACCATCACACC
Human	Mdr1	CTTGTTAGACAGCCTCATA	TCATACAGTCAGAGTTCAC
	PXR	CTGTGTGGATGCTGAGCTGT	TGATTGTCAGCGTAGCCTTG
	PPARα	TTCGCAATCCATCGGCGAG	CCACAGGATAAGTCACCGAGG
	β-actin	AGCGAGCATCCCCCAAAGTT	GGGCACGAAGGCTCATCATT

Table S2. Input parameters utilized in the PBPK model[22]

Parameters		Unit	Value
		Dabigatran	
Molecular weight		g/mol	471.51
Solubility		mg/mL	0.017
LogP		-	2.2
Solubility		mg/mL	0.017
pK_a		-	4.1（base）<br>4.4（base）<br>12.4（base）
Blood:plasma ratio		-	0.67
Fraction unbound (%)		-	65
P_{app}		cm/s	0.08×10^{-5}
CL_{CR}-healthy subjects		mL/min	103
CL_{CR}-mild reanal impairment		mL/min	67
CL_{CR}-moderate reanal impairment		mL/min	41
Enzyme Table			
UGT2B15	K_m	μmol/L	512
	V_{max}	pmol/min/mg	31.8
	abundance	pmol/mg	62.1
		Dabigatran etexilate	
Molecular weight		g/mol	627
LogP		-	5.17
pK_a		-	4.0（acid）<br>6.7（base）
Solubility		mg/mL	1.8
Blood:plasma ratio		-	3.08
Fraction unbound (%)		-	7
P_{app}		cm/s	1.4×10^{-4}
Enzyme Table			
CES 1	K_m	μmol/L	24.9
	V_{max}	pmol/min/mg	676.0
	abundance	pmol/mg	1664.4
CES 2	K_m	μmol/L	5.5
	V_{max}	pmol/min/mg	71.1
	abundance	pmol/mg	174.1
Transporter Table			
P-gp	K_m	μmol/L/	38.9
Gut	V_{max}	pmol/min/million	146

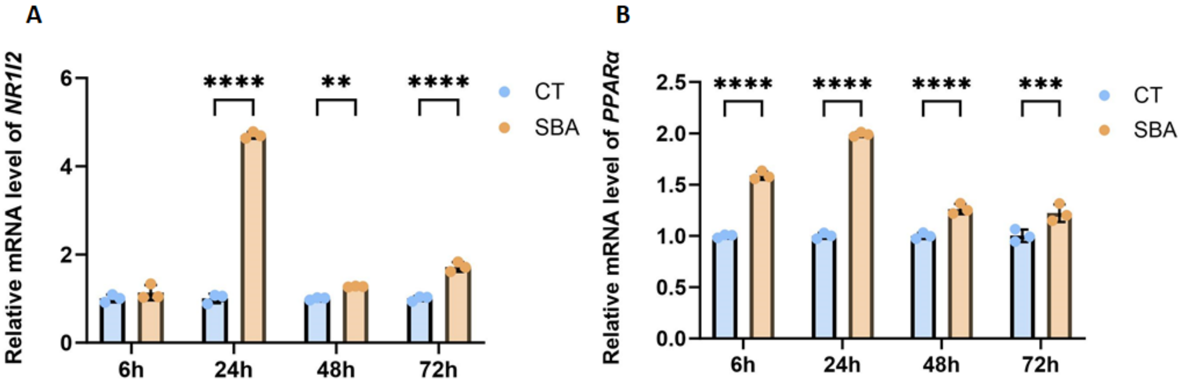

Figure S1. Relative mRNA expression of NR1I2 (A) and PPARα (B) following treatment with or without physiologically relevant concentrations of SBA for 6, 24, 48, and 72 hours.

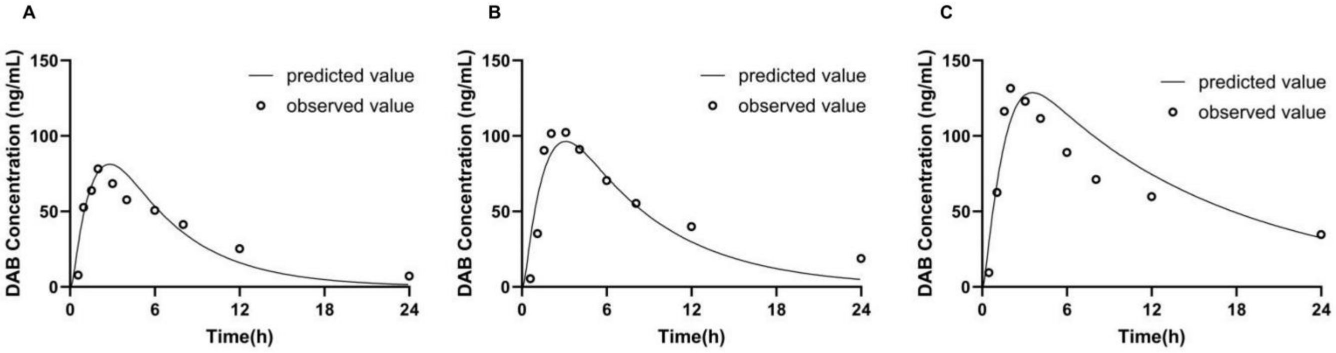

Figure S2. Validation of the DABE-DAB PBPK model in (A) healthy controls, (B) patients with mild renal impairment, and (C) patients with moderate renal impairment following a single 150 mg dose of DABE[32]
