# Supplementary material for: Moxifloxacin-Mediated Downregulation of Intestinal P-Glycoprotein Alters the Pharmacokinetics of Dabigatran Etexilate: Mechanistic Insights in Rats and PBPK Model-Informed Dose Optimization
Source: Pharmaceutics. 2026 Aug 20;18(8):1031. doi: 10.3390/pharmaceutics18081031 (PMC13516782; doi:10.3390/pharmaceutics18081031)
Supplement: Supplementary file 1 [file pharmaceutics-18-01031-s001.zip › Supplemental Materials S2.pdf]

LC-MS/MS method for determination of dabigatran in rat serum

1. Methods

1.1 Calibration curves & quality control samples

The 200 μg/mL dabigatran stock solutions in DMSO were diluted with methanol to obtain intermediate solutions at a concentration of 100 μg/mL. These intermediate solutions were then used to prepare mixed working solutions for the calibration curves, which were subsequently diluted with blank rat serum to produce serum calibration standards and serum quality control (QC) samples. The final serum concentrations of the calibration standards were 5, 12.5, 25, 75, 250, 750, 2500, 7500, and 25000 ng/mL. The lower limit of quantitation (LLOQ) was set at 5 ng/mL, with low, middle, and high QC concentrations of 12.5, 1500, and 20000 ng/mL, respectively. All calibrator and QC samples were stored at －80° C. Quantification was performed using d4-rivaroxaban as the internal standard (IS), and the peak area of each analyte was normalized against that of the IS compound.

1.2 Sample Preparation

Calibration curve samples, QC samples, and frozen rat serum samples to be analyzed were thawed at room temperature. An aliquot of 50 μL of serum was transferred to a 1.5-mL Eppendorf tube, followed by the addition of 100 μL of IS working solution (10 μg/mL in 100% acetonitrile). The mixture was vortexed for 1 min and centrifuged at 18,000×g for 10 min at 4°C. The resulting supernatant was collected for subsequent LC-MS/MS analysis.

1.3 Chromatographic and mass spectrometry conditions

In this project, the separation of the target compounds was performed on a Waters XTerra RP18 column (4.6×150 mm, 5 μm) using an API4000 system (AB Sciex, USA) operated in positive ion mode. The MS/MS parameters for DAB were as follows: MS/MS transition, m/z 472.5(the precursor ion)/289.1(the product ion); declustering potential, 84 V; and collision energy, 36 V. For the IS, the transition was m/z 440.4/145.3, with the declustering potential set at 95 V and the collision energy at 37 V. The mobile phase contained 5 mM aqueous ammonium acetate (A) and acetonitrile (B). The mobile phase flow rate was 0.6 mL/min and injection volume was 10 μL. The gradient program was as follows: 30% A from 0 to 3.8 min, 5% A at 4 min, 5% A from 4 to 5.5min, and 30% A from 5.6 to 8 min. Acquisition was performed in multiple reaction monitoring (MRM) mode.

1.4. Method validation

Method validation followed standard guidelines for bioanalytical method development. Selectivity and specificity assays were carried out by assessing six different blank serum samples and monitoring for potential chromatographic interferences from endogenous substances. Calibration curves for each analyte were

calculated by linear regression with $1/x^2$ weighting within respective linearity ranges. Intra- and inter-day precisions were validated by repeating the analysis on the same day (intra-day precision, n = 6) and on three consecutive days (inter-day precision, n = 18). The extraction recovery and matrix effect were assessed by analyzing QC samples at low, middle and high concentrations, respectively.

2. Results

2.1 Selectivity

Blank serum samples from six different rats were taken and analyzed, the results suggested that no endogenous interference was observed within the retention times of dabigatran (**Figure S3A**). The retention time of dabigatran and IS was 2.19 min and 3.01 min (**Figure S3B-C**).

2.2 Linearity, precision and accuracy

In this linearity validation, three standard curves prepared were evaluated in parallel. The calibration curve equations, correlation coefficient and the linearity range of the the analyte were listed in **Table S3**, suggesting favorable linearity of the dabigatran within the linearity range of the rat serum. The precision and accuracy data at four concentration levels (LLOQ, LQC, MQC, and HQC) are presented in **Table S4**. The results demonstrate that this analysis method meet generally accepted requirements for quantitative analysis of biological samples.

2.2 Recovery and matrix effect

The extraction recoveries of dabigatran at low, middle, and high QC concentrations were 94.89% ± 3.76%, 105.22% ± 4.07%, and 98.90% ± 1.24%, respectively. The corresponding matrix effects were 87.77% ± 3.54%, 94.08% ± 3.67%, and 90.98% ± 1.77%, respectively. These results demonstrate that the extraction and detection procedures for this analyte are reliable and acceptable.

2.3 Stability

The stability test was determined with dabigatran in rat serum under different storage conditions, including the short-term stability (at room temperature for 4 h), long-term stability (at -80 ℃ for one month), freeze/thaw stability (-80 ℃ to room temperature in a triple cycle), and autosampler stability (4 ℃, 6 h). The stability data are presented in **Table S5**. The results suggested that no significant degradation was observed under different storage conditions, and the data obtained in these storage conditions are accurate and reliable.

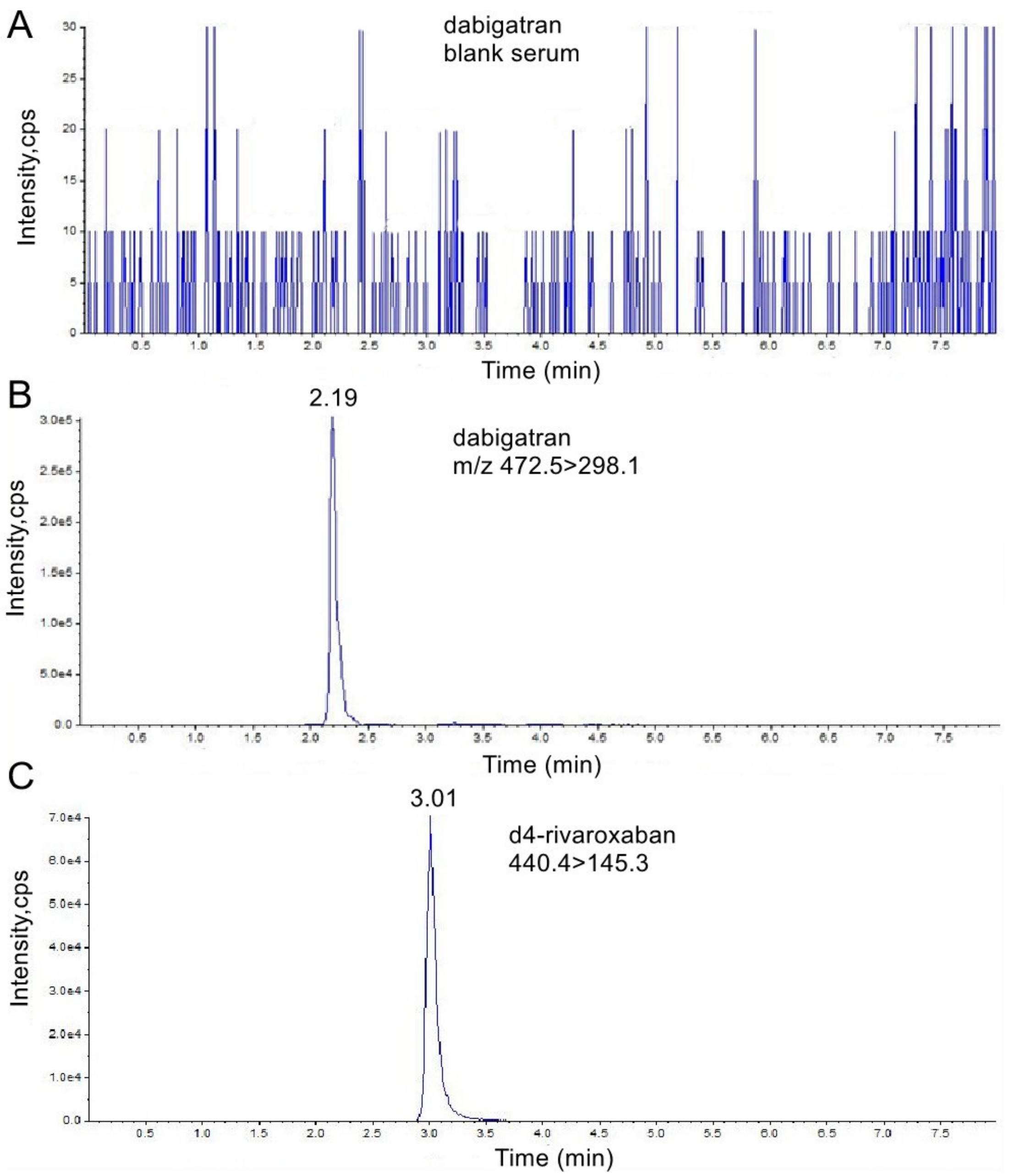

Figure S3. Chromatograms of blank serum (A), dabigatran-spiked serum (B), and IS-spiked serum (C).

Table S3. Calibration curve equations, correlation coefficient and the linearity range of dabigatran in rat serum samples.

Analyte	Linear equation	Correlation coefficient (r^2)	Linearity range (ng/mL)
dabigatran	$Y=2.07\times10^{-4}X+7.89\times10^{-5}$	0.9985	0.5-2500

Table S4. Intra-day and inter-day precision and accuracy of dabigatran in rat serum samples.

Analyte	Theoretical concentration (ng/mL)	Intra-day (n = 6)		Inter-day (n = 6)	
		RSD(%)	RE(%)	RSD(%)	RE(%)
dabigatran	0.5	10.50	-0.48	7.16	3.77
	1.25	6.53	1.02	6.09	1.47
	150	6.22	-7.19	4.35	-4.67
	2000	13.58	-4.56	1.97	-6.08

Table S5. Stability of dabigatran in rat serum samples.

Analyte	Theoretical concentration (ng/mL)	Room temperature for 4 h	Autosampler for 6 h	three freeze-thaw	-80 ℃ for 1 month
		(%, mean ± SD)			
dabigatran	1.25	96.90±4.42	98.93±7.93	93.67±5.32	88.70±1.71
	150	103.33±3.06	98.87±5.60	97.63±0.96	88.17±1.15
	2000	95.50±0.72	101.67±0.58	88.40±2.01	90.73±5.60
